# A Review of Psoriasis, a Known Risk Factor for Cardiovascular Disease and Its Impact on Folate and Homocysteine Metabolism

**DOI:** 10.1155/2012/965385

**Published:** 2012-05-29

**Authors:** Ian McDonald, Maureen Connolly, Anne-Marie Tobin

**Affiliations:** Department of Dermatology, The Adelaide and Meath Hospital Incorporating the National Children's Hospital, Tallaght, Dublin 24, Ireland

## Abstract

Psoriasis is a chronic inflammatory skin condition with an increased risk of cardiovascular disease. This risk has been attributed to an association with many independent risk factors including obesity, hypertension, smoking, and dyslipidemia. Psoriasis patients also have lower levels of folate and conversely higher levels of homocysteine, which in itself is a risk factor for cardiovascular disease. It has been postulated that low folate levels in this group may be a direct cause of hyperhomocysteinemia and therefore a treatable risk factor by folate supplementation. This paper looks at the literature published to date on the relationship between psoriasis, homocysteine, and folate levels.

## 1. Introduction

Psoriasis is a chronic recurrent inflammatory skin condition. Its prevalence varies among ethnic groups, but it affects approximately 1–3% [1] of the population in industrialised countries. Its clinical course can vary greatly in terms of morphology, distribution, and severity of the disease. In its commonest subtype it is characterised by the formation of discrete, pink, scaly plaques occurring at various sites on the body. It affects men and women equally and is seen in all races [2]. Although it can present at any age, it occurs most frequently between the ages of 15 and 20 and again between 50 and 60 years of age.

Psoriasis has very significant psychosocial morbidity which appears independent of objective disease severity [3, 4]. It is also associated with an increased risk of cardiovascular disease and mortality [5–8]. Indeed patients with psoriasis have almost twice the risk of cardiovascular disease when compared with normal controls [9, 10]. The exact reason for this increased risk is unknown. A number of studies have shown significantly increased rates of hypertension, dyslipidemia, diabetes mellitus, smoking, and excessive alcohol consumption in patients with psoriasis [11]. Neimann et al. found that patients with severe psoriasis had an increased risk of diabetes (odds ratio (OR), 1.62; 95% confidence interval (CI), 1.3–2.01), obesity (OR, 1.79; 95% CI, 1.55–2.05), and smoking (OR, 1.31; 95% CI, 1.17–1.47) compared with controls [12]. Prodanovich et al. also found a higher prevalence of these risk factors in patients with psoriasis. However, even after controlling for these variables, they found a higher prevalence of ischemic heart disease (OR 1.78; 95% CI, 1.51–2.11), cerebrovascular disease (OR, 1.70; 95% CI, 1.33–2.17), and peripheral vascular disease (OR, 1.98; 95% CI, 1.32–2.82) when compared with controls. Indeed they also found psoriasis to be an independent risk factor for mortality (OR, 1.86; 95% CI, 1.56–2.21) as a result of the association with atherosclerosis [13].

 These risk factors in combination with the chronic inflammatory process are thought to be significant components in the development of vascular disease [9, 14]. Links with alterations in levels of folate and homocysteine in patients with psoriasis have also been implicated in contributing to the propagation of atherosclerosis and atherothrombotic events [9, 15, 16].

## 2. Pathogenesis of Low Folate and High Homocysteine Levels in Patients with Psoriasis

 Recent case control studies have demonstrated that patients with psoriasis have lower levels of folate in comparison to normal controls [9, 15, 17, 18]. The exact aetiology of this association remains unclear. Postulated mechanisms include alterations in gut absorption of folate due to microscopic inflammatory changes seen in the bowel mucosa of patients with active psoriasis and psoriatic arthritis [15]. A more likely explanation however probably relates to the accelerated keratinocyte turnover seen in patients with psoriasis. This action results in excessive consumption of folate used to methylate DNA in these actively dividing cells thus lowering folate levels [9].

Conversely homocysteine levels are elevated in psoriasis patients [9, 15, 17]. In one case-controlled study this was found to directly correlate with disease severity and to be inversely related to plasma folate levels [15]. Plasma homocysteine is an independent risk factor for cardiovascular disease [19, 20], peripheral vascular disease, cerebrovascular disease and possibly Alzheimer's diseases. The magnitude of this risk is equivalent to the risk of smoking or dyslipidemia [9].

**Figure 1:**
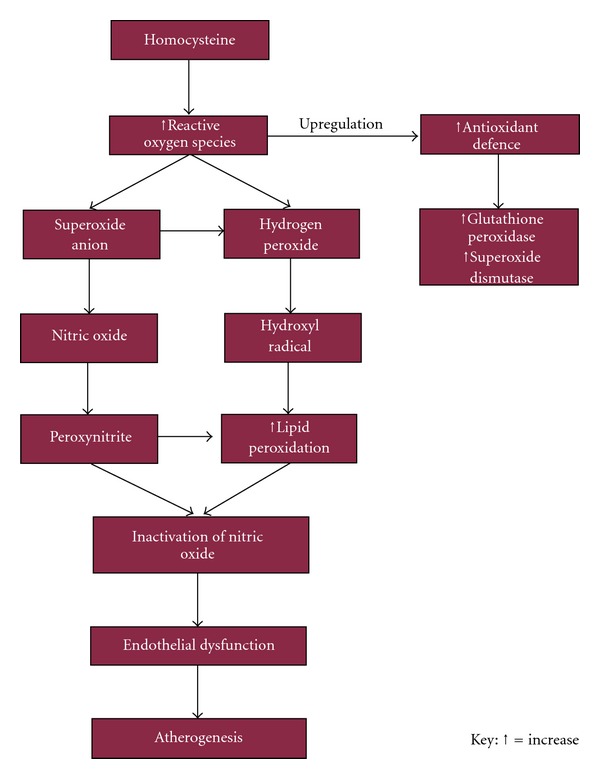
Proposed mechanism of homocysteine-induced endothelial dysfunction and atherogenesis [21].

 Hyperhomocysteinemia (>15 umol/L) is thought to favour atherosclerosis and vascular thrombosis by a number of mechanisms. These include damaging endothelial cells, promoting clot formation, decreasing flexibility of blood vessels leading to aortic stiffness, and reducing blood flow velocity [20]. The endothelial dysfunction is thought to result from the accumulation of asymmetrical dimethylarginine (ADMA) which is a natural inhibitor of nitric oxide synthase. As a result there is a reduction in the production of the vasodilator nitric oxide which protects the vessel wall against the pathogenesis of atherosclerosis and thrombosis (Figure 1).

It has been suggested that hyperhomocysteinemia in addition to other factors may be caused by reduced levels of folate in these patients [9, 15]. Coenzymes methylene tetra-hydrofolate, methylcobalamin, and pyridoxal phosphate are essential for three of the enzymes involved in the metabolism of homocysteine and are dependent on folate, vitamin B12 and B6, respectively. Hence in patients with severe psoriasis who have large areas of rapid skin turnover and increased keratinocyte activity, there is excessive consumption of folate. This in turn results in reduced breakdown and elevated serum levels of homocysteine with all of its adverse effects.

## 3. Discussion

 It appears from this paper that patients with psoriasis have lower levels of folate and higher levels of homocysteine than normal controls. As psoriasis is associated with an increased risk of cardiovascular morbidity and mortality and homocysteine is an independent risk factor for cardiovascular disease, it may seem intuitive that managing this risk factor would have beneficial effects in terms of cardiovascular mortality and morbidity. 

**Figure 2:**
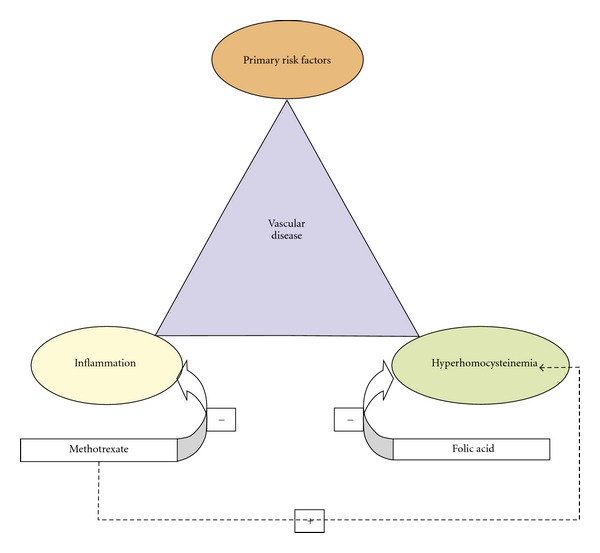
Proposed action of combination therapy, in addition to risk factors such as age, sex, hypertension, and dyslipidemia, homocysteine and inflammation lead to increased incidence of vascular disease. Consequently reducing inflammation with methotrexate and hyperhomocysteinemia by folic acid may lead to a decreased incidence of vascular disease for patients with psoriasis [24].

 The possible benefit of folate supplementation seems logical [22]. Folate has long been used in combination with methotrexate in the management of psoriasis, psoriatic arthritis, and rheumatoid arthritis. Here it is effective in reducing gastrointestinal side effects and liver function test abnormalities [23]. In a retrospective cohort study looking at over seven thousand patients with psoriasis, methotrexate was found to reduce the incidence of vascular disease and this reduction was further enhanced when folic acid was added (Figure 2) [24].

However, homocysteine levels can be elevated for other reasons including obesity, hypertension, smoking, and excessive alcohol consumption all of which have significant prevalence in the psoriatic patient population [11]. Armitage et al. in a double-blinded randomised control trial assessed the beneficial effects of lowering homocysteine levels in over 12,000 post-MI patients. They failed to demonstrate any benefit in vascular outcomes from long-term reductions in homocysteine with folate and vitamin B12 supplementation [25]. It is possible that hyperhomocysteinemia in psoriasis is independent of folate deficiency and instead is linked with other risk factors such as hypertension and obesity [9]. “The psoriatic march”, a concept of how severe psoriasis may drive cardiovascular morbidity, suggests a process of genetic susceptibility triggered by environmental factors and immune responses. This leads to disease expression and co-morbidity resulting from chronic inflammation. It is this chronic inflammatory burden and state of insulin resistance which can result in endothelial dysfunction and ultimately atherosclerosis [26].

## 4. Conclusion

 It is well established that folic acid supplementation has a role in the treatment of psoriasis in conjunction with methotrexate treatment. However, the evidence to support its use in reducing the risk of cardiovascular disease by directly impacting on plasma homocysteine levels is lacking. Certainly further work within this group is necessary. For now management of associated risk factors such as obesity, dyslipidemia, and hypertension as well as encouraging smoking cessation are paramount for these patients as is, of course, the management of their skin.
